# Biopsychosocial Variables in Male Schizophrenic Patients: A Comprehensive Comparison with Healthy Controls

**DOI:** 10.3390/ph16121633

**Published:** 2023-11-21

**Authors:** Krzysztof Krysta, Beata Trędzbor, Ewa Martyniak, Aleksandra Cieślik, Agnieszka Koźmin-Burzyńska, Katarzyna Piekarska-Bugiel, Katarzyna Skałacka, Rafał Bieś, Marek Krzystanek

**Affiliations:** 1 Department and Clinic of Psychiatric Rehabilitation, Faculty of Medical Sciences, Medical University of Silesia, Ziołowa 45/47, 40-635 Katowice, Poland; beataziarko@poczta.onet.pl (B.T.); evamartyniak@gmail.com (E.M.); krzystanekmarek@gmail.com (M.K.); 2 Departament of Neurological and Psychiatric Nursing, Chair of Neurology, School of Health Sciences in Katowice, Medical University of Silesia, Ziołowa 45/47, 40-635 Katowice, Poland; acieslik@sum.edu.pl; 3 Department of Psychiatric Rehabilitation, Leszek Giec Upper-Silesian Medical Centre, Medical University of Silesia, Ziołowa 45/47, 40-635 Katowice, Poland; kozminburzynska@gmail.com (A.K.-B.); kpiekarskabugiel@gmail.com (K.P.-B.); 4 Institute of Psychology, University of Opole, Plac Staszica 1, 45-052 Opole, Poland; katarzyna.skalacka@uni.opole.pl; 5 Medical Students’ Association, Department and Clinic of Psychiatric Rehabilitation, Faculty of Medical Sciences, Medical University of Silesia, Ziołowa 45/47, 40-635 Katowice, Poland; s82700@365.sum.edu.pl

**Keywords:** schizophrenia, anthropometric measurements, biochemical markers, psychopathology, lifestyle factors, pharmacological assessment, cognitive function

## Abstract

Objective: this study aims to comprehensively compare neuropsychological, psychopathological, anthropometric, biochemical, pharmacological, and lifestyle variables between 27 male schizophrenic patients (SZ group) and 30 age- and sex-matched healthy male controls (HC group). Methods: participants underwent a battery of neuropsychological tests including the Trail Making Test (TMT), Stroop Color-Word Interference Test, and Verbal Fluency Test. Psychopathological symptoms in the SZ group were evaluated using the Positive and Negative Syndrome Scale (PANSS). Anthropometric measurements such as body weight, height, BMI, and waist circumference were taken. Biochemical markers measured included fasting glucose, total cholesterol, triglycerides, high-density lipoprotein (HDL) cholesterol, low-density lipoprotein (LDL) cholesterol, and fasting insulin. Lifestyle factors were assessed through a questionnaire for the study of views and eating habits of people aged 16 to 65. Results: the HC group outperformed the SZ group in the TMT_A test and the Stroop test, but no significant differences were observed in the TMT_B test or in phonemic fluency tests. No correlation was found between age and PANSS scores within the SZ group. Anthropometrically, the SZ group had higher body weight, waist circumference, and BMI, with no difference in height. Biochemically, the HC group had higher HDL cholesterol levels but lower insulin and insulin resistance indices. Pharmacological assessment showed a more significant impact on body weight among SZ patients taking second-generation antipsychotics. Lifestyle factors such as diet and screen time were comparable between groups, but the SZ group reported longer sleep duration and lower leisure time activity. Conclusions: our study highlights distinct neuropsychological, pharmacological, anthropometric, and biochemical differences between male schizophrenic patients and healthy controls. The results underscore the complexity of schizophrenia and point toward the need for a multi-faceted approach to its management and understanding.

## 1. Introduction

This study is driven by pivotal research questions aimed at understanding the complex relationship between schizophrenia and its biopsychosocial implications. Our primary objective is to investigate the extent to which male schizophrenic patients differ from healthy male controls in neuropsychological, psychopathological, anthropometric, biochemical, pharmacological, and lifestyle aspects. Specifically, we seek to ascertain the persistence of cognitive and metabolic impairments in schizophrenic patients despite antipsychotic medication, exploring the potential resistance to treatment. By examining these variables, our study endeavors to shed light on whether the enduring symptoms of schizophrenia are a direct consequence of the disease pathology or an outcome of chronic medication use. The purpose of this research is to contribute to a more nuanced understanding of schizophrenia, thereby aiding in the development of more effective and comprehensive therapeutic strategies.

### 1.1. Schizophrenia: A Multifaceted Mental Health Condition

Schizophrenia is a complex mental health condition featuring a range of symptoms, including significant cognitive impairments. Recent research has increasingly highlighted the association between metabolic syndrome (MetS) and cognitive dysfunction in patients with this disorder [1,2]. A broader meta-analysis has expanded this understanding, examining the impact of cardiovascular risk factors, including MetS, diabetes, and hypertension, on cognitive outcomes in schizophrenia [2]. Metabolic syndrome (MetS) shows a particularly heightened prevalence among those diagnosed with schizophrenia, reaching rates that are nearly twice as high as those in the general population [3,4,5]. While antipsychotic medications are pivotal for treating schizophrenia, they often worsen MetS and its components, including diabetes, hypertension, and hyperlipidemia [5,6,7].

### 1.2. Impact of Antipsychotic Medications on Metabolic Syndrome

Conflicting evidence exists regarding the impact of antipsychotic polypharmacy on MetS in schizophrenia, as highlighted by a 2018 review by Ijaz et al. Some combinations, particularly those involving aripiprazole, may confer protective effects against diabetes and hyperlipidemia as compared to other antipsychotic combinations and monotherapies [6]. Second-generation antipsychotics, as studied by Hasnain et al. (2010) and Riordan et al. (2011), are associated with elevated risks of MetS in schizophrenia patients. These medications differentially impact weight gain, waist circumference, fasting triglyceride levels, and glucose levels [5,7]. Clozapine and olanzapine are noted to have higher risks [4,5]. Riordan et al. (2011) underscore the need for regular clinical monitoring of weight, symptoms of hyperglycemia, and glucose levels in patients undergoing chronic antipsychotic treatment. Despite these recommendations, adherence to monitoring remains limited due to lack of awareness, resource constraints, and organizational challenges [5].

### 1.3. Cardiovascular Risk Factors and Cognitive Dysfunction in Schizophrenia

The existing literature identifies cardiovascular risk factors—most notably those encapsulated within MetS—as key determinants of cognitive dysfunction among individuals afflicted with schizophrenia [8]. The seminal meta-analysis by Hagi et al. (2021) meticulously evaluated 27 studies, encompassing 10,174 participants diagnosed with schizophrenia. This rigorous investigation unequivocally demonstrated a substantial link between cardiovascular risk factors and global cognitive impairment. Specifically, individuals with schizophrenia who also presented with metabolic syndrome (MetS) exhibited pronounced cognitive deficits, evidenced by an effect size (ES) of 0.31 (*p* = 0.001). This impact was similarly significant among those with diabetes (ES = 0.32; *p* < 0.001) and hypertension (ES = 0.21; *p* < 0.001). Additionally, these patients displayed compromised performance in discrete cognitive domains [2]. Such revelations corroborate prior research, underscoring the pivotal role that MetS and related cardiovascular factors play in exacerbating cognitive deficits observed in schizophrenia [1]. Both Hagi et al. (2021) and Schmitt et al. (2018) illuminate the intricate dynamics between cardiovascular risk elements and cognitive capabilities within the schizophrenia spectrum. As a prospective therapeutic avenue, physical exercise interventions, particularly those focused on aerobic activities, have shown promise in ameliorating these cardiovascular risk variables while simultaneously enhancing cognitive function [2,8]. Given this connection between cardiovascular risk factors and cognitive impairment, the role of exercise in managing schizophrenia becomes particularly relevant. Bueno-Antequera and Munguía-Izquierdo (2020) emphasize that physical activity and exercise offer promising avenues for both prevention and treatment [9]. 

The anticipation of further high-caliber clinical trials promises to deepen our understanding of these complex interactions, paving the way for more targeted and personalized treatment paradigms for individuals grappling with schizophrenia. Bora et al.’s 2017 systematic review and Battini et al.’s 2023 study both point to a significant association between MetS and cognitive impairment in schizophrenia. The studies encompass metabolic components such as hypertension, dyslipidemia, abdominal obesity, and diabetes [1,10]. Zeng et al. (2021) introduce the gut microbiota as a potential intermediary between MetS and cognitive deficits in schizophrenia. This angle broadens the scope of the conversation, suggesting that antipsychotic medications could interact with gut microbiota and add further complexity to metabolic and cognitive outcomes [11]. The work by Battini et al. highlights the promise of metformin, a drug well-known for treating MetS, in potentially enhancing cognitive function in schizophrenia [10]. This body of research aligns with the review by MacKenzie et al. (2018), which focuses on the intricate connections between antipsychotic drugs, MetS, and cognitive function [12]. Antipsychotic medications can exacerbate metabolic issues, further contributing to cognitive decline, as indicated by both Bora et al. and Battini et al. [1,10].

### 1.4. Emerging Research: Gut Microbiota and Molecular Interactions

Emerging research has been focusing on the intricate molecular interactions between metabolic syndrome (MetS), cognitive deficits, and schizophrenia [13,14]. An important advancement in this field has been the elucidation of the gut microbiota’s role as a vital intermediary that connects MetS with cognitive issues within the schizophrenia context [11]. This interaction takes place through a complex interplay of neuronal, endocrine, and immunological mechanisms collectively known as the gut–brain axis. The use of second-generation antipsychotics introduces another layer of complexity. These medications can interact with and potentially alter the gut microbiota, thereby inducing or exacerbating the adverse effects associated with MetS and cognitive impairments in schizophrenia patients [11]. Previous studies have examined the gut microbiota’s regulatory effects on psychiatric conditions like anxiety and depression. However, only a few have specifically investigated its role in second-generation antipsychotics-induced MetS and cognitive dysfunctions. Adding to this, second-generation antipsychotics such as olanzapine and clozapine are notorious for posing a high metabolic risk, affecting systems like the hypothalamus, liver, pancreatic β-cells, and adipose tissue [13]. They act on dopamine, serotonin, acetylcholine, and histamine receptors, affecting neuropeptides and 5’AMP-activated protein kinase (AMPK) activity, leading to metabolic imbalances [13].

In response to these challenges, novel therapeutic interventions are swiftly being developed. For instance, prebiotics and probiotics have emerged as microbiota-management tools with proven efficacy for treating metabolic imbalances and cognitive deficits [11]. Concurrently, a multidisciplinary approach involving psychoeducation and therapeutic drug monitoring is being considered as a frontline strategy to mitigate the risks of second-generation antipsychotics-associated MetS [13].

### 1.5. Integrative Treatment Paradigm: Addressing Metabolic Syndrome in Schizophrenia

Metabolic syndrome (MetS) is not a peripheral issue in the context of schizophrenia; rather, it is fundamentally intertwined with the disorder’s complex pathology [15]. This intricate linkage necessitates an integrative treatment paradigm that encompasses not only psychiatric care but also addresses the metabolic challenges inherent to schizophrenia. Previous studies have highlighted the essential roles of early intervention, rigorous metabolic monitoring, and judicious pharmacological choices with minimal metabolic side effects [16]. Additionally, non-pharmacological interventions like specialized exercise programs and diet modifications have been proposed as valuable tools to offset the negative impact of MetS on cognitive outcomes in schizophrenia patients [15]. While the importance of a multi-disciplinary approach is increasingly recognized, a knowledge gap still persists concerning the relationship between MetS and cognitive functions in schizophrenia patients. The existing literature underscores the complexity of antipsychotic medications and their propensity to contribute to MetS, especially in the realm of polypharmacy [5,6,7]. However, the interaction between MetS and cognitive functions within this patient group remains inadequately explored.

In this vein, the current study seeks to address this gap. The aim of this study is to elucidate how MetS correlates with cognitive functions in schizophrenia patients. Additionally, the study will evaluate the clinical importance of systematic metabolic screening and the implementation of supportive programs. This study aims also to offer evidence-based insights that can inform and enrich the current multi-disciplinary approaches to treating schizophrenia, thus emphasizing the need for a comprehensive, integrated model of care. 

### 1.6. Theoretical Framework

Our research is underpinned by the biopsychosocial model, which views schizophrenia as a complex interplay of biological, psychological, and social factors. This framework is crucial in examining the convergence of metabolic syndrome (MetS) and cognitive dysfunction in schizophrenia. It highlights the necessity for an integrated treatment approach, considering the resistance to current treatments in addressing cognitive deficits and metabolic side effects. By acknowledging these multifaceted impacts, our study advocates for a holistic treatment strategy that addresses the intricate nature of schizophrenia.

## 2. Results

### 2.1. Neuropsychological Assessment

In the TMT_A test, participants from the control group (healthy group) received significantly fewer points than participants from the clinical group, but in the TMT_B test, there were no statistically significant differences between groups. Detailed results are presented in Table 1.

In both versions of the Stroop test, participants from the control group (healthy group) received significantly fewer points than participants from the clinical group. Detailed results are presented in Table 1.

In all tests related to phonemic fluency, the conducted analyses showed no significant differences between the control group and the clinical group. In tests relating to semantic fluency, the analyses carried out showed that the subjects in the control group received more points than those in the clinical group. The last semantic fluency test is an exception, where no significant differences were found between the control group and the clinical group. Detailed results are presented in Table 1.

### 2.2. Psychopathology Assessment

The conducted correlation analysis in the clinical group showed no relationship between age and the level of symptoms from the PANSS test. 

### 2.3. Anthropometric Measurements

The analysis of anthropometric parameters showed that people from the clinical group had significantly higher body weight, waist circumference, and BMI than people from the control group (healthy). The subjects from the clinical group do not differ significantly from the subjects from the control group in height. Detailed results are presented in Table 2.

### 2.4. Biochemical Markers

In the case of biochemical indices, participants from the control group had significantly higher HDL cholesterol parameters, but, at the same time, significantly lower insulin levels and lower insulin resistance indices than participants from the clinical group. Other biochemical indices did not differ in the level among men from both study groups. The conducted analysis shows that the levels of glucose, total cholesterol, HDL cholesterol, LDL cholesterol, triglycerides, insulin, and HOMA-IR do not significantly correlate with the results of any of the cognitive tests. 

### 2.5. Pharmacological Assessment

In the case of the effect of drugs on body weight, the chi^2^ test showed that among patients taking atypical drugs, they are significantly more often associated with the effect on their body weight than among patients taking classic drugs. The stronger the drug’s impact on body weight, the lower the insulin levels were found. The effect size is moderate (Rho = −0.44). Other laboratory parameters are not associated with the drug’s impact on body weight. 

### 2.6. Diet and Lifestyle Assessment

Index of healthy/unhealthy diet: the level of parameters related to the diet does not differ significantly between the subjects from the control and clinical groups. 

Lifestyle: in the case of lifestyle, smoking cigarettes currently or in the past, the number of hours in front of the TV/computer, and activity undertaken on working days did not differentiate the clinical group from the control group. On the other hand, the number of hours of sleep on working days and weekends is significantly higher in the clinical group than in the control group, but leisure time activity is significantly higher in the control group than in the clinical group. Detailed data are presented in Table 3.

## 3. Discussion

### 3.1. Key Findings

Our study revealed significant differences in neuropsychological performance, anthropometric measurements, and biochemical markers between male schizophrenic patients and healthy controls. These differences are consistent with the recently published findings of Huang et al. (2023), who investigated the association between metabolic risk factors and cognitive impairment in schizophrenia [17]. Higher scores in TMT and Stroop tests among the SZ group indicate worse neuropsychological performance, mirroring the observations of Cao et al. (2023) regarding the impact of metabolic syndrome on cognitive function and the benefits of psychological interventions [18]. The biochemical markers showed significant variations in metabolic health between the two groups. Elevated insulin levels and higher HOMA-IR indices in the SZ group could suggest a link between schizophrenia and metabolic syndromes like insulin resistance. Interestingly, no differences were noted in fasting glucose or LDL cholesterol levels, meaning that the metabolic abnormalities may be more specific rather than generalized. The significantly higher HDL cholesterol in the HC group also points toward a healthier metabolic profile compared to the SZ group.

### 3.2. Contextualization within Existing Literature

In terms of cognitive performance, our finding of higher scores in TMT and Stroop tests among the SZ group aligns with the general observation of impaired cognitive performance in schizophrenic populations, as also noted by Lindenmayer et al. (2012), Goughari et al. (2015), and others [19,20]. These tests indicate that schizophrenia patients experience impairments in areas such as psychomotor speed, visuospatial working memory, and verbal working memory, confirming previously established patterns of cognitive deficits in this population.

Our study stands out in its examination of biochemical markers alongside cognitive measures. Elevated insulin levels and higher HOMA-IR indices in the SZ group resonate with the findings of Luckhoff et al. (2019), who also pointed out metabolic abnormalities in a population of first-episode schizophrenia patients [21]. However, unlike other studies that found more generalized metabolic issues, our results hint at a more specific set of metabolic disturbances, particularly around insulin resistance. This specificity could be instrumental in tailoring treatments targeting metabolic health to improve cognitive outcomes. 

It is noteworthy that our study did not find a difference in fasting glucose or LDL cholesterol levels between the groups. This suggests that not all aspects of metabolic syndrome may be relevant or have the same kind of relationship with cognitive functioning in schizophrenia, which is consistent with the nuanced view provided by Goughari et al. (2015) [20].

In relation to antipsychotic medication, we found that those taking atypical antipsychotics were more likely to experience weight gain, confirming the observations of Haddad (2005) and Sussman (2003) [22,23]. We also observed a counterintuitive finding that men on antipsychotics who gained weight had lower insulin levels, an aspect that calls for more targeted research. 

### 3.3. Antropomorphic Measures 

In our study, we found significant differences in anthropometric parameters, such as body weight, waist circumference, and BMI, between the clinical and control groups. These findings contribute to the complex landscape of antipsychotic-related metabolic effects. When comparing our results to the existing literature, several studies offer relevant insights. For instance, Hansen et al. (2016) also reported small but significant increases in BMI and waist circumference among patients with schizophrenia [24]. Similarly, a study by Zhang et al. (2020) assessed the metabolic impacts of seven different antipsychotic drugs and found significant changes in BMI and waist circumference, among other metabolic measures [25].

### 3.4. Antipsychotic Treatment 

Our study adds to the growing body of research on the metabolic implications of antipsychotic medications, revealing that patients taking atypical antipsychotics are more likely to experience effects on their body weight compared to those taking classic drugs. Incorporating the findings of our study with those from previous research provides a nuanced understanding of weight changes associated with antipsychotic medications. Our study supports the observation that weight gain differs significantly among patients taking different types of antipsychotic drugs, both atypical and typical. While Hrdlicka et al. (2009) conducted a retrospective study focusing on adolescents, they found that atypical antipsychotics (AAP) led to a more rapid weight gain in the first week of treatment compared to typical antipsychotics (TAP). However, this difference was not sustained in subsequent weeks, a point that aligns with our results, suggesting that the initial rate of weight gain may not be indicative of long-term trends [26]. Haddad (2005) and Sussman (2003) both focused on weight gain in adult populations and emphasized the role of specific atypical antipsychotics, like clozapine and olanzapine, in significant weight gain [22,23]. These findings complement our study by highlighting that the implications of antipsychotic-associated weight gain can vary depending on the population studied (adolescent versus adult) and the duration of medication use. 

In our study, we also observed a correlation between treatment with antipsychotics that have a higher potential for increasing body weight and lower insulin levels. This is an intriguing result, as it seems counterintuitive at first glance. Weight gain is usually associated with increased insulin resistance, which often leads to higher circulating insulin levels, not lower. The observed correlation could suggest that the mechanisms by which these antipsychotic drugs cause weight gain might differ from traditional pathways, such as overeating or lack of physical activity. Perhaps these medications are affecting metabolic rate, appetite regulation, or directly altering insulin sensitivity in ways that are not yet fully understood. Generally, weight gain is associated with increased insulin resistance and higher insulin levels [27]. However, our study could propose a discussion about the complex layer that exists in our understanding of the metabolic effects related to antipsychotic treatment. When viewed alongside the cellular insights from Kowalchuk et al. (2019) and the meta-analytical data from Burghardt et al. (2018), our findings cautiously suggest the need for a more nuanced understanding of the effects of antipsychotic medication [27,28]. Additionally, our observations on weight gain and insulin levels contribute to a broader understanding of antipsychotic side effects, which Abbas et al. (2023) suggest could be mitigated by incorporating exercise into treatment regimes [29].

### 3.5. Resistance to Current Treatments

In contemplating the differential variables between patients with schizophrenia and control subjects, it is crucial to consider the resistance to current treatments observed in clinical practice. Our findings reveal that despite the administration of antipsychotic medication, certain metabolic and cognitive impairments persist, suggesting a potential resistance to treatment. For example, while antipsychotics are effective in managing positive symptoms of schizophrenia, they appear less effective in reversing cognitive deficits or preventing metabolic side effects such as weight gain and insulin resistance. This resistance to treatment underscores the necessity for developing a broader spectrum of therapeutic interventions. It also raises the question of whether these persistent symptoms are a direct consequence of the disease pathology or result from chronic medication use. Understanding the nature of these treatment-resistant variables is paramount in designing comprehensive care strategies.

### 3.6. Lifestyle Factors

In our study, we found no significant differences between the clinical and control groups when it came to lifestyle choices such as current or past smoking habits, the amount of time spent in front of screens, or levels of activity on workdays. However, the clinical group did report significantly more hours of sleep on both weekdays and weekends compared to the control group. Conversely, the control group was more active during leisure time than the clinical group. In synthesizing our findings with prior research, our study complements the work performed, among others, by Heald et al. (2017), Gurusamy et al. (2018), and Flocco et al. (2023), but offers distinct insights [30,31,32]. In contrast to the existing literature, our study provides a nuanced view of lifestyle factors and metabolic health in individuals with mental illnesses. For example, Flocco et al. (2023) focused on reducing sedentary behavior through targeted intervention in an inpatient setting [32], Gurusamy et al. (2018) concentrated on the effectiveness of diet and exercise interventions for metabolic syndrome in schizophrenia [31], and Heald et al. (2017) explored the prevalence of metabolic syndrome and lifestyle factors in a UK-based outpatient population with schizophrenia [30]. Our research diverges significantly in that we did not find a meaningful difference in sedentary behaviors such as TV/computer time or workday activity levels between the clinical and control groups. However, we did observe higher sleep durations in the clinical group and greater leisure time activity in the control group. These findings suggest that lifestyle factors may not be the sole contributors to metabolic health in our population and bring into question the universal applicability of intervention strategies.

### 3.7. Limitations

Our paper has several limitations. Firstly, the study’s exclusive focus on male participants limits the generalizability of its findings, as schizophrenia manifests differently between sexes, with varying symptomatology, treatment responses, and risk factors. This limitation is consistent with the literature emphasizing the importance of including both sexes in schizophrenia research to capture sex-specific nuances [33].

Secondly, the study’s cross-sectional design prevents the establishment of causal relationships between variables. While cross-sectional studies are suitable for examining associations, they cannot uncover causation. Therefore, the study’s findings may not shed light on the directional relationships between the studied variables.

Lastly, the study’s assessment of biochemical measures, though comprehensive to some extent, omitted potentially relevant markers. This oversight underscores the need for future research to conduct a more thorough evaluation of biochemical variables associated with schizophrenia and its comparison with healthy controls.

### 3.8. Therapeutic Implications and Predictions

Our research has illuminated a spectrum of neuropsychological, biochemical, and anthropometric variances between schizophrenic patients and healthy individuals, which are presented in Table 4. These findings suggest several avenues for therapeutic exploration. Neuropsychological assessments demonstrated that schizophrenic patients often exhibit deficits in psychomotor speed, visuospatial working memory, and verbal working memory, which indicates potential targets for cognitive enhancement therapies. Moreover, the relationship between schizophrenia and metabolic abnormalities, particularly insulin resistance, suggests that treatments extending beyond traditional antipsychotic medication may be required. This includes potential interventions focusing on metabolic health, such as dietary regulation and lifestyle changes to manage weight gain—an often-observed side effect of antipsychotic medication. The data also revealed lifestyle factors that are intrinsically linked to the well-being of patients with schizophrenia. These include increased sleep duration and decreased leisure activity, which may offer novel intervention targets. For instance, lifestyle modification strategies, including sleep hygiene education and engagement in regular physical activity, could be integrated into treatment plans. The introduction of such interventions should be considered carefully, given their implications for personalizing patient care and the possibility of resistance to current treatments.

### 3.9. Implications for Future Research

Our study confirms that people with schizophrenia perform poorly on certain cognitive tests. Future research should focus on longitudinal studies to observe how these cognitive abilities change over time and whether different treatment methods can improve them. We found significant differences in body weight, waist circumference, and BMI between schizophrenic patients and healthy controls. The next step should be to investigate the underlying mechanisms behind these differences to answer the question if it is due to the medications, lifestyle, or both, and whether targeted interventions, like specific diets or drugs, can mitigate these differences [9,13,34]. Our study shows that atypical antipsychotic medications are more likely to result in weight gain compared to classical antipsychotics. This prompts the need for further studies to understand why this occurs and how it can be managed. Comparative studies on the metabolic effects of individual antipsychotic drugs would be beneficial. The role of lifestyle factors in metabolic health among people with schizophrenia needs to be further examined. It would be useful to understand whether interventions aimed at lifestyle changes can have a positive impact on both metabolic health and psychiatric symptoms. In summary, our study opens up several avenues for future research to provide a more comprehensive understanding of schizophrenia, particularly in terms of cognitive function, body measurements, medication effects, insulin levels, and lifestyle factors. We also invite future researchers to employ Online Photovoice (OPV), Outcome Indicator Photovoice Assessment (OIPA), and Community-Based Participatory Research (CBPR) for an in-depth analysis of schizophrenia [35]. OPV allows individuals to portray their schizophrenia experience, using visual stories to supplement quantitative findings. Its effectiveness is well-documented across different studies, such as Doyumgaç et al.’s (2021) [36], which examined online education challenges during the pandemic, Subasi’s (2023) [37] work on university students’ sense of belonging, and Tanhan & Strack’s (2020) [38] study on the biopsychosocial spiritual wellbeing of Muslims. For a holistic understanding of schizophrenia, future studies might investigate the subjective impact of treatment modalities, incorporating diverse methodologies that reflect the multifaceted nature of patient wellbeing.

## 4. Materials and Methods

At the core of our study lies a comparative cross-sectional research design, aimed at elucidating the neuropsychological, psychopathological, anthropometric, biochemical, pharmacological, and lifestyle differences between male schizophrenic patients and healthy male controls. This design facilitates a comprehensive and systematic examination of the multifaceted aspects of schizophrenia. By recruiting participants from a day rehabilitation program and the local community, and employing a range of diagnostic tools and assessments, our study provides an in-depth exploration of the complex interplay between various biopsychosocial factors and schizophrenia.

### 4.1. Subjects

The study sample consisted of 27 male schizophrenic patients (SZ group) aged 29 to 48 years and 30 age- and sex-matched healthy male controls (HC group), aged 21 to 59 years. Schizophrenic patients were recruited from a day rehabilitation program of the Department and Clinic of Psychiatric Rehabilitation in the Leszek Giec Upper-Silesian Medical Centre, Katowice, and the healthy controls were recruited from the local community. The inclusion criteria for the SZ group were a clinical diagnosis of schizophrenia based on the ICD-10 criteria [39] and an age between 18 and 60 years. The exclusion criteria for both groups included any history of neurological disorders, substance abuse, or other medical conditions that may influence cognitive function or metabolic parameters. Written informed consent was obtained from all participants.

### 4.2. Neuropsychological Assessment

All participants underwent neuropsychological assessment using the following tests:

- Trail Making Test (TMT)—part A and part B [40], which measured psychomotor speed (part A) and visuospatial working memory (part B).
- Stroop Color-Word Interference Test—part RCNb and part NCWd [41], which assessed reading speed (part RCNb) and verbal working memory (part NCWd).
- Verbal Fluency Test—categorical and phonological [42], which evaluated semantic and phonemic fluency.

### 4.3. Psychopathology Assessment

The severity of psychopathological symptoms in the SZ group was assessed using the Positive and Negative Syndrome Scale (PANSS) [43].

### 4.4. Anthropometric Measurements

Height and weight were measured using a medical scale, and body mass index (BMI) was calculated as weight (kg) divided by height squared (m^2^). Waist circumferences were measured using anthropometric tape [44].

### 4.5. Biochemical Markers

Blood samples were collected from participants after an overnight fast. The following biochemical markers were measured: fasting glucose, total cholesterol, triglycerides, high-density lipoprotein (HDL) cholesterol, low-density lipoprotein (LDL) cholesterol, and fasting insulin [45]. The Homeostatic Model Assessment for Insulin Resistance (HOMA-IR) index was calculated [46].

### 4.6. Pharmacological Assessment

For the SZ group, information on current antipsychotic medication including equivalent chlorpromazine dose [47] and the use of typical versus second-generation antipsychotics was collected from medical records. We also included an analysis of antipsychotic medication according to the risk of developing metabolic syndrome, based on data from the Polish translation of the Psychotropic Drug Directory [48].

### 4.7. Diet and Lifestyle Assessment

All participants completed the “Kwestionariusz do badania poglądów i zwyczajów żywieniowych dla osób w wieku od 16 do 65 lat, version 1.1” (in Polish) (questionnaire for the study of views and eating habits of people aged 16 to 65, version 1.1) [49], which assessed dietary habits, nutritional knowledge, and lifestyle factors such as sleep, smoking, physical activity, and hours spent in front of a computer.

### 4.8. Ethical Approval

The study protocol was approved by the Bioethics Committee of the Medical University of Silesia, with the decision number PCN/0022/KB1/134/19.

### 4.9. Statistical Analysis

The data were analyzed with IBM SPSS STATICTICS v.29. Descriptive analyses aimed to identify the basic level of the variable analyzed. In the case of variables where a lack of normal distribution was noted, differences were calculated with the U Mann–Whitney test and Kruskal–Wallis ANOVA test. Moreover, the chi^2^ test, V-Kramer test, and Spearman’s correlation coefficient were used to determine the relationships between the variables without normal distribution. In the case of variables meeting the assumption of normal distribution, a *t*-student test and Pearson r correlation coefficient were used. Missing data were omitted in all the analyses, with statistical significance set at 0.05.

## 5. Conclusions

In conclusion, our study contributes to the growing body of evidence on the multi-dimensional challenges faced by patients with schizophrenia. By considering both cognitive and metabolic markers, we offer a more comprehensive understanding of the disease. Future research should aim to validate these findings in larger, more diverse populations and to explore the complex interactions between these markers in shaping the course and outcomes of schizophrenia.

## Figures and Tables

**Table 1:** Differences between control and clinical group in neuropsychological parameters.

	GROUP	N	Mean	SD	Significance ^a^
**TMT_A**	**1**	**30**	**31.42**	**13.822**	**U = 191.50; Z = −2.50; *p* = 0.012**
	**2**	**27**	**45.11**	**20.554**	
TMT_B	1	30	82.00	37.482	U = 223.00; Z = −1.91; *p* = 0.057
	2	27	99.93	41.252	
Mistakes	1	30	2.61	7.095	U = 303.00; Z = −0.21; *p* = 0.834
	2	27	1.15	2.597	
**STROOP1**	**1**	**30**	**14.87**	**3.209**	**U = 130.00; Z = −3.53; *p* < 0.001**
	**2**	**27**	**20.22**	**6.518**	
**STROOP2**	**1**	**30**	**26.30**	**7.564**	**U = 151.00; Z = −3.11; *p* = 0.002**
	**2**	**27**	**37.74**	**19.827**	
Mistakes	1	30	0.57	1.199	U = 298.50; Z = −2.50; *p* = 0.012
	2	27	0.59	1.010	
Phonemic fluency	1	30	9.83	3.298	t= −0.38; df = 48; *p* = 0.709
	2	27	10.22	4.041	
Phonemic fluency	1	30	11.78	4.242	t = −0.45; df = 48; *p* = 0.661
	2	27	12.37	5.039	
Phonemic fluency	1	30	11.35	3.676	t = 0.51; df = 48; *p* = 0.614
	2	27	10.78	4.182	
**Semantic fluency**	**1**	**30**	**20.00**	**5.099**	**t = 3.09; df = 48; *p* = 0.003**
	**2**	**27**	**15.70**	**4.705**	
**Semantic fluency**	**1**	**30**	**14.48**	**2.921**	**t = 3.12; df = 48; *p* = 0.003**
	**2**	**27**	**11.67**	**3.374**	
Semantic fluency	1	30	11.48	2.906	t = 0.925; df = 48; *p* = 0.360
	2	27	10.44	4.635	

Note: 1—control group; 2—clinical group; a—in case of variables where lack of normal distribution was noted, differences were calculated with U Mann–Whitney test; otherwise, it was a *t*-student test; bolded results are statistically significant.

**Table 2:** Difference in anthropometric parameters between control and clinical group.

	Group	N	Mean	SD	Significance ^a^
**Body weight (kg)**	**1**	**30**	**82.33**	**15.094**	**t = −3.23; df = 55; *p* = 0.002**
	**2**	**27**	**96.11**	**17.068**	
Height (cm)	1	30	176.10	9.437	t = −0.33; df = 55; *p* = 0.747
	2	27	176.96	10.639	
**Waist circumference (cm)**	**1**	**30**	**93.50**	**10.312**	**t = −3.73; df = 55; *p* < 0.001**
	**2**	**27**	**105.70**	**14.250**	
**BMI**	**1**	**30**	**26.4947**	**4.30493**	**t = −3.34; df = 55; *p* < 0.001**
	**2**	**27**	**30.9333**	**5.69595**	

Note: 1—control group; 2—clinical group; a—*t*-test was used to calculate the differences between groups; bolded results are statistically significant.

**Table 3:** Lifestyle differences between the control and clinical groups.

		Group		Together	
		1	2		Significance ^a^
**Cigarettes now (0—no; 1—yes)**	**0**	23	14	37	Chi^2^ = 3.84; df = 1; *p* = 0.050
	1	7	13	20	
Together		30	27	57	
Cigarettes in the past (0—no; 1—yes)	0	14	8	22	Chi^2^ = 1.74; df = 1; *p* = 0.187
	1	16	19	35	
Together		30	27	57	
**Hours of sleep—workdays (1–3)**	**1**	**10**	**1**	**11**	**V = 0.58; df = 2;** ***p*** **< 0.001**
	**2**	**20**	**15**	**35**	
	**3**	**0**	**11**	**11**	
Together		30	27	57	
**Hours of sleep—weekend (1–3)**	**1**	**4**	**1**	**5**	**V = 0.51; df = 2;** ***p*** ** < 0.001**
	**2**	**20**	**7**	**27**	
	**3**	**6**	**19**	**25**	
**Together**		**30**	**27**	**57**	
Hours at TV/computer (1–6)	1	6	3	9	V = 0.387; df = 5; *p* = 0.130
	2	9	14	23	
	3	6	6	12	
	4	1	3	4	
	5	6	1	7	
	6	2	0	2	
Together		30	27	57	
Activity on workdays (1–3)	1	14	12	26	V = 0.204; df = 2; *p* = 0.312
	2	7	10	17	
	3	9	4	13	
Together		30	26	56	
Activity in free time (1–3)	1	1	13	14	**V = 0.52; df = 2;** ***p*** **< 0.001**
	2	22	11	33	
	3	7	3	10	
Together		30	27	57	

Note: 1—control group; 2—clinical group; a—significance was calculated with a chi^2^ test or V-Kramer test; bolded results are statistically significant.

**Table 4:** Therapeutic implications and predictions.

Variable	Group Difference	Clinical Implication
Neuropsychological performance	Worse in SZ group indicated by TMT and Stroop test scores	Potential for cognitive enhancement therapies targeting psychomotor speed and working memory deficits
Anthropometric measurements	Higher body weight, waist circumference, and BMI in SZ group	Need for weight management and metabolic health interventions
Biochemical markers	Elevated insulin levels and HOMA-IR indices in SZ group suggest metabolic syndrome link	Insight into antipsychotic treatment effects on metabolic health; insulin resistance as a treatment focus
Lifestyle factors	Longer sleep duration and lower leisure time activity in SZ group	Consideration for lifestyle modification strategies in therapeutic regimes

## Data Availability

Data are contained within the article.

## References

[1] Bora E., Akdede B.B., Alptekin K. (2017). The relationship between cognitive impairment in schizophrenia and metabolic syndrome. Psychol. Med..

[2] Hagi K., Nosaka T., Dickinson D., Lindenmayer J.P., Lee J., Friedman J., Boyer L., Han M., Abdul-Rashid N.A., Correll C.U. (2021). Association Between Cardiovascular Risk Factors and Cognitive Impairment in People with Schizophrenia: A Systematic Review and Meta-analysis. JAMA Psychiatry.

[3] Mitchell A.J., Vancampfort D., Sweers K., van Winkel R., Yu W., De Hert M. (2013). Prevalence of metabolic syndrome and metabolic abnormalities in schizophrenia and related disorders--a systematic review and meta-analysis. Schizophr. Bull..

[4] Vancampfort D., Stubbs B., Mitchell A.J., De Hert M., Wampers M., Ward P.B., Rosenbaum S., Correll C.U. (2015). Risk of metabolic syndrome and its components in people with schizophrenia and related psychotic disorders, bipolar disorder and major depressive disorder: A systematic review and meta-analysis. World Psychiatry.

[5] Riordan H.J., Antonini P., Murphy M.F. (2011). Atypical antipsychotics and metabolic syndrome in patients with schizophrenia: Risk factors, monitoring, and healthcare implications. Am. Health Drug Benefits.

[6] Ijaz S., Bolea B., Davies S., Savović J., Richards A., Sullivan S., Moran P. (2018). Antipsychotic polypharmacy and metabolic syndrome in schizophrenia: A review of systematic reviews. BMC Psychiatry.

[7] Hasnain M., Fredrickson S.K., Vieweg W.V., Pandurangi A.K. (2010). Metabolic syndrome associated with schizophrenia and atypical antipsychotics. Curr. Diabetes Rep..

[8] Schmitt A., Maurus I., Rossner M.J., Röh A., Lembeck M., von Wilmsdorff M., Takahashi S., Rauchmann B., Keeser D., Hasan A. (2018). Effects of Aerobic Exercise on Metabolic Syndrome, Cardiorespiratory Fitness, and Symptoms in Schizophrenia Include Decreased Mortality. Front. Psychiatry.

[9] Bueno-Antequera J., Munguía-Izquierdo D. (2020). Exercise and Schizophrenia. Adv. Exp. Med. Biol..

[10] Battini V., Cirnigliaro G., Leuzzi R., Rissotto E., Mosini G., Benatti B., Pozzi M., Nobile M., Radice S., Carnovale C. (2023). The potential effect of metformin on cognitive and other symptom dimensions in patients with schizophrenia and antipsychotic-induced weight gain: A systematic review, meta-analysis, and meta-regression. Front. Psychiatry.

[11] Zeng C., Yang P., Cao T., Gu Y., Li N., Zhang B., Xu P., Liu Y., Luo Z., Cai H. (2021). Gut microbiota: An intermediary between metabolic syndrome and cognitive deficits in schizophrenia. Prog. Neuropsychopharmacol. Biol. Psychiatry.

[12] MacKenzie N.E., Kowalchuk C., Agarwal S.M., Costa-Dookhan K.A., Caravaggio F., Gerretsen P., Chintoh A., Remington G.J., Taylor V.H., Müeller D.J. (2018). Antipsychotics, Metabolic Adverse Effects, and Cognitive Function in Schizophrenia. Front. Psychiatry.

[13] Carli M., Kolachalam S., Longoni B., Pintaudi A., Baldini M., Aringhieri S., Fasciani I., Annibale P., Maggio R. (2021). Atypical Antipsychotics and Metabolic Syndrome: From Molecular Mechanisms to Clinical Differences. Pharmaceuticals.

[14] Molina J.D., Avila S., Rubio G., López-Muñoz F. (2021). Metabolomic Connections between Schizophrenia, Antipsychotic Drugs and Metabolic Syndrome: A Variety of Players. Curr. Pharm. Des..

[15] Doménech-Matamoros P. (2020). Influence of the use of atypical antipsychotics in metabolic syndrome. Rev. Esp. Sanid. Penit..

[16] Pramyothin P., Khaodhiar L. (2010). Metabolic syndrome with the atypical antipsychotics. Curr. Opin. Endocrinol. Diabetes Obes..

[17] Huang H., Du L., Pu Z., Shi Y., Xiao Z., Chen X., Yao S., Wang L., Li Z., Xue T. (2023). Association between Metabolic Risk Factors and Cognitive Impairment in Schizophrenia Based on Sex. Psychiatry Investig..

[18] Cao A., Tang C., Chen X., Li C. (2023). Analysis of Metabolic Syndrome and Cognitive Functional Analysis in Schizophrenic Patients Based on Psychological Intervention. Prev. Med..

[19] Lindenmayer J.P., Khan A., Kaushik S., Thanju A., Praveen R., Hoffman L., Cherath L., Valdez G., Wance D. (2012). Relationship between metabolic syndrome and cognition in patients with schizophrenia. Schizophr. Res..

[20] Goughari A.S., Mazhari S., Pourrahimi A.M., Sadeghi M.M., Nakhaee N. (2015). Association of metabolic syndrome with cognitive function in patients with schizophrenia. J. Psychiatr. Res..

[21] Luckhoff H.K., Kilian S., Olivier M.R., Phahladira L., Scheffler F., du Plessis S., Chiliza B., Asmal L., Emsley R. (2019). Relationship between changes in metabolic syndrome constituent components over 12 months of treatment and cognitive performance in first-episode schizophrenia. Metab. Brain Dis..

[22] Haddad P. (2005). Weight change with atypical antipsychotics in the treatment of schizophrenia. J. Psychopharmacol..

[23] Sussman N. (2003). The implications of weight changes with antipsychotic treatment. J. Clin. Psychopharmacol..

[24] Hansen M.V., Hjorth P., Kristiansen C.B., Vandborg K., Gustafsson L.N., Munk-Jørgensen P. (2016). Reducing cardiovascular risk factors in non-selected outpatients with schizophrenia. Int. J. Soc. Psychiatry.

[25] Zhang Y., Wang Q., Reynolds G.P., Yue W., Deng W., Yan H., Tan L., Wang C., Yang G., Lu T. (2020). Metabolic Effects of 7 Antipsychotics on Patients with Schizophrenia: A Short-Term, Randomized, Open-Label, Multicenter, Pharmacologic Trial. J. Clin. Psychiatry.

[26] Hrdlicka M., Zedkova I., Blatny M., Urbanek T. (2009). Weight gain associated with atypical and typical antipsychotics during treatment of adolescent schizophrenic psychoses: A retrospective study. Neuro Endocrinol. Lett..

[27] Burghardt K.J., Seyoum B., Mallisho A., Burghardt P.R., Kowluru R.A., Yi Z. (2018). Atypical antipsychotics, insulin resistance and weight; a meta-analysis of healthy volunteer studies. Prog. Neuropsychopharmacol. Biol. Psychiatry.

[28] Kowalchuk C., Kanagasundaram P., Belsham D.D., Hahn M.K. (2019). Antipsychotics differentially regulate insulin, energy sensing, and inflammation pathways in hypothalamic rat neurons. Psychoneuroendocrinology.

[29] Abbas M.S., Nassar S.T., Tasha T., Desai A., Bajgain A., Ali A., Dutta C., Pasha K., Paul S., Venugopal S. (2023). Exercise as an Adjuvant Treatment of Schizophrenia: A Review. Cureus.

[30] Heald A., Pendlebury J., Anderson S., Narayan V., Guy M., Gibson M., Haddad P., Livingston M. (2017). Lifestyle factors and the metabolic syndrome in Schizophrenia: A cross-sectional study. Ann. Gen. Psychiatry.

[31] Gurusamy J., Gandhi S., Damodharan D., Ganesan V., Palaniappan M. (2018). Exercise, diet and educational interventions for metabolic syndrome in persons with schizophrenia: A systematic review. Asian J. Psychiatry.

[32] Flocco P., Pompili E., Riggio F., Nicolò G., Bernabei L. (2023). Men.Phys—Reducing sedentary behavior and increasing physical activity in people with severe mental illness in an acute psychiatric ward: A research protocol for a randomized controlled trial. Clin. Ter..

[33] Li Q., Chen D., Liu T., Walss-Bass C., de Quevedo J.L., Soares J.C., Zhao J., Zhang X.Y. (2016). Sex Differences in Body Mass Index and Obesity in Chinese Patients with Chronic Schizophrenia. J. Clin. Psychopharmacol..

[34] Adamowicz K., Mazur A., Mak M., Samochowiec J., Kucharska-Mazur J. (2020). Metabolic Syndrome and Cognitive Functions in Schizophrenia-Implementation of Dietary Intervention. Front. Psychiatry.

[35] Dari T., Fox C., Laux J.M., Speedlin Gonzalez S. (2023). The Development and Validation of the Community-Based Participatory Research Knowledge Self-Assessment Scale (CBPR-KSAS): A Rasch Analysis. Meas. Eval. Couns. Dev..

[36] Doyumgaç I., Tanhan A., Kiymaz M.S. (2021). Understanding the Most Important Facilitators and Barriers for Online Education during COVID-19 through Online Photovoice Methodology. Int. J. High. Educ..

[37] Subasi Y. (2023). College Belonging among University Students during COVID-19: An Online Interpretative Phenomenological (OIPA) Perspective. J. Happiness Health.

[38] Tanhan A., Strack R.W. (2020). Online Photovoice to Explore and Advocate for Muslim Biopsychosocial Spiritual Wellbeing and Issues: Ecological Systems Theory and Ally Development. Curr. Psychol..

[39] WHO (1992). The ICD-10 Classification of Mental and Behavioural Disorders: Clinical Descriptions and Diagnostic Guidelines.

[40] Reitan R.M. (1958). Validity of the Trail Making Test as an indicator of organic brain damage. Percept. Mot. Skills.

[41] Stroop J.R. (1935). Studies of interference in serial verbal reactions. J. Exp. Psychol..

[42] Borkowski J.G., Benton A.L., Spreen O. (1967). Word fluency and brain damage. Neuropsychologia.

[43] Kay S.R., Fiszbein A., Opler L.A. (1987). The positive and negative syndrome scale (PANSS) for schizophrenia. Schizophr. Bull..

[44] WHO (2011). Waist Circumference and Waist-Hip Ratio: Report of a WHO Expert Consultation, Geneva, 8–11 December 2008.

[45] Expert Panel on Detection, Evaluation, and Treatment of High Blood Cholesterol in Adults (2001). Executive Summary of the Third Report of the National Cholesterol Education Program (NCEP) Expert Panel on Detection, Evaluation, and Treatment of High Blood Cholesterol in Adults (Adult Treatment Panel III). JAMA.

[46] Matthews D.R., Hosker J.P., Rudenski A.S., Naylor B.A., Treacher D.F., Turner R.C. (1985). Homeostasis model assessment: Insulin resistance and beta cell function in man. Diabetologia.

[47] Woods S.W. (2003). Chlorpromazine equivalent doses for the newer atypical antipsychotics. J. Clin. Psychiatry.

[48] Bazire S. (2018). Przewodnik Leków Psychotropowych 2018—Tom 2: Podręcznik dla Lekarzy i Podsumowanie Najważniejszych Informacji.

[49] Jeżewska-Zychowicz M., Gawęcki J., Wądołowska L., Gawęcki J. (2014). i wsp. Kwestionariusz do badania poglądów i zwyczajów żywieniowych dla osób w wieku od 16 do 65 lat, wersja 1.1—Kwestionariusz administrowany przez ankietera-badacza. Kwestionariusz do Badania Poglądów i Zwyczajów Żywieniowych oraz Procedura Opracowania Danych.

